# Significance of somatic mutations and content alteration of mitochondrial DNA in esophageal cancer

**DOI:** 10.1186/1471-2407-6-93

**Published:** 2006-04-18

**Authors:** Duan-Jun Tan, Julia Chang, Ling-Ling Liu, Ren-Kui Bai, Yu-Fen Wang, Kun-Tu Yeh, Lee-Jun C Wong

**Affiliations:** 1 Institute for Molecular and Human Genetics, Georgetown University Medical Center, Washington DC, USA; 2 Department of Pathology, Changhua Christian Hospital, ChangHua, Taiwan; 3 Department of Molecular and Human Genetics, Baylor College of Medicine, Houston, Texas 77030, USA

## Abstract

**Background:**

The roles of mitochondria in energy metabolism, the generation of ROS, aging, and the initiation of apoptosis have implicated their importance in tumorigenesis. In this study we aim to establish the mutation spectrum and to understand the role of somatic mtDNA mutations in esophageal cancer.

**Methods:**

The entire mitochondrial genome was screened for somatic mutations in 20 pairs (18 esophageal squamous cell carcinomas, one adenosquamous carcinoma and one adenocarcinoma) of tumor/surrounding normal tissue of esophageal cancers, using temporal temperature gradient gel electrophoresis (TTGE), followed by direct DNA sequencing to identify the mutations.

**Results:**

Fourteen somatic mtDNA mutations were identified in 55% (11/20) of tumors analyzed, including 2 novel missense mutations and a frameshift mutation in ND4L, ATP6 subunit, and ND4 genes respectively. Nine mutations (64%) were in the D-loop region. Numerous germline variations were found, at least 10 of them were novel and five were missense mutations, some of them occurred in evolutionarily conserved domains. Using real-time quantitative PCR analysis, the mtDNA content was found to increase in some tumors and decrease in others. Analysis of molecular and other clinicopathological findings does not reveal significant correlation between somatic mtDNA mutations and mtDNA content, or between mtDNA content and metastatic status.

**Conclusion:**

Our results demonstrate that somatic mtDNA mutations in esophageal cancers are frequent. Some missense and frameshift mutations may play an important role in the tumorigenesis of esophageal carcinoma. More extensive biochemical and molecular studies will be necessary to determine the pathological significance of these somatic mutations.

## Background

The human mitochondrial genome is a circular double stranded DNA of 16.6 kb encoding 13 respiratory chain protein subunits, 22 tRNAs, and 2 rRNAs. The mitochondrial mRNA contains no introns. There is a 1.2-kb hypervariable non-coding D-loop region that is susceptible to somatic DNA mutations. Each cell contains hundreds to thousands of mitochondria, and each mitochondrion contains 2–10 copies of mitochondrial DNA (mtDNA) [1]. The major function of mitochondria is to produce energy to support cellular activities through the oxidative phosphorylation pathway. During this process reactive oxygen species (ROS) are generated. Due to the lack of protective histone proteins and the close vicinity, mtDNA is an easy target for oxidative DNA damage by ROS. In addition, the limited DNA repair mechanism allows mtDNA mutations to accumulate. Thus, the mutation rate of mtDNA is at least 10 times higher than that of nuclear DNA. The roles of mitochondria in energy metabolism, the generation of ROS, aging, and the initiation of apoptosis have implicated their importance in tumorigenesis [2].

Neoplastic transformation is a multi-step process in that alterations in multiple nuclear genes have been extensively documented. Somatic mitochondrial DNA (mtDNA) changes during tumorigenesis have also been recognized in recent years [3-13]. However, unlike the common mtDNA mutations in maternally inherited mitochondrial disease, the functional significance and pathogenic mechanism of somatic mtDNA mutations in cancer development remains unclear despite the vast evidence of their occurrence in various types of tumors [3-8,10-13]. The identification of tumor suppressing functions of several genes that are involved in energy metabolism [14-18] and the role of the mitochondria in apoptotic pathways [19], have suggested that mtDNA alterations might be an important integral of tumorigenesis and programmed cell death. Evidences of down-regulation of bioenergetic function of mitochondria have been documented [20,21].

Extensive analysis of the mitochondrial genome using direct sequencing has revealed that approximately 30–70% of all types of tumors harbor mtDNA alterations [3-8,10,12,13,22,23]. A majority of these studies focused on the analysis of hypervariable, non-coding D-loop region [4,5,10,22-24]. Comprehensive mutational analysis of the entire mitochondrial genome achieved by direct sequencing of approximately 80% of the mitochondrial genome [3,6] or by the use of TTGE mutation screening method with overlapping primers covering the entire genome was limited to only a few studies [7,8,11-13]. Previous reports [3,6] demonstrated that most of the somatic mtDNA mutations found in cancer were in the homoplasmic form. This observation led to the conclusion that mutant mitochondria gained a replicative advantage during tumorigenesis and became homoplasmic within a few generations [3]. Our recent investigation of somatic mtDNA mutations in breast, oral, and brain tumors revealed that mutations in the coding region did occur and there were significant number of heteroplasmic alterations [7,8,13].

Esophageal cancer is one of the most common and aggressive cancers in Central and Southeast China, including Taiwan [25-27]. A high incidence rate of >1.25 per 1,000 and a cumulative mortality rate of 20–25% have been reported [25,26]. Somatic mtDNA mutations in the non-coding D-loop region occurred in 5 and 34% of primary esophageal tumors reported by two Japanese studies [4,28]. A similar investigation of Chinese patients from Shanxi province also reported D-loop mtDNA mutations in 33% of esophageal tumors [26]. These studies were limited to the non-coding hypervariable region only. Results from others and our studies of various types of cancers suggest that the somatic mtDNA mutation spectrum may vary among different tissues [3,4,6-8,10-13]. Furthermore, unlike the mutations in the non-coding region, novel missense and frameshift mutations in coding region may have pathogenic significance [11,12]. In addition, changes in mtDNA content in tumors may have clinicopathological implication in tumorigenesis [12]. In this report, we use TTGE mutation scanning method to study somatic mtDNA mutations in the entire mitochondrial genome of 20 esophageal tumors and their surrounding tissue. We also evaluated the mtDNA content in the tumor and its surrounding tissue and correlated the molecular findings with the clinicopathological profile of the tumor.

## Methods

### Tissue specimens and DNA extraction

Paired tumors and surrounding tissues were previously banked specimens that were surgically removed from patients with histologically confirmed esophageal cancer (18 esophageal squamous cell carcinomas, one adenosquamous carcinoma and one adenocarcinoma). The age of the patients (1 female and 19 male) were from 38 to 73 years old with the mean age of 59 ± 14. The specimens were obtained from the tumor bank of the Pathology Department of Changhua Christian Hospital, Changhua, Taiwan, according to an institutionally approved protocol. DNA was isolated from frozen tissues using proteinase K digestion and phenol/chloroform extraction.

### Comprehensive mutational analysis of the entire mitochondrial genome

Comprehensive mutation analysis of the entire mitochondrial genome by temporal temperature gradient gel electrophoresis (TTGE) has been described previously [7,29,30]. Thirty-two pairs of overlapping primers were used to amplify the entire 16.6-kb mitochondrial genome [29]. The DNA fragments vary in size from 306-bp to 805-bp with an average of 594-bp [7,29,30]. Each fragment has an average of 74-bp on each end overlapping with the neighboring fragments. PCR products were denatured at 95°C for 30 s and slowly cooled to 45°C for a period of 45 min at a rate of 1.1°C/min. TTGE was performed on a Bio-Rad D-Code apparatus. The polyacrylamide (acrylamide:bisacrylamide 37.5:1) gels were prepared in 1.25 × Tris-Acetate-EDTA buffer containing 6 mol/L urea. Electrophoresis was carried out at 135 V for 4–6 h at a constant temperature increment of 1–2°C/h. The beginning and ending temperature were determined by computer simulation from the melting curve (50% denatured) of the DNA fragment [7,29,30]. On TTGE analysis, a single band shift represents a homoplasmic DNA alteration, and a multiple-banding pattern represents a heteroplasmic mutation. The DNA fragments from tumor and surrounding tissue of the same patient were analyzed side-by-side.

### Identification of mutations by DNA sequencing

Any DNA fragments showing differences in banding patterns between tumors and surrounding samples were sequenced to identify the exact mutations. The DNA sequencing was performed by using the big dye terminator cycle sequencing kit (Perkin Elmer) and an ABI 377 (Applied Biosystems) automated sequencer. The results of DNA sequence analysis were compared with the complete human mitochondrial sequence deposited in GenBank (accession number NC_001807) ; [31] using MacVector 7.0 (Oxford Molecular Ltd, Oxford, England) software. Any DNA sequence differences between tumor and matched normal mtDNA were scored as somatic mutations. Sequence variations found in both tumor and matched normal mtDNA but different from that recorded in the GenBank were scored as germline variations. Those not recorded in MitoMap database were categorized as novel mtDNA variations, and those appear in the database were categorized as reported polymorphisms or mutations.

### Measurement of mtDNA contents in tumor and surrounding tissue

The mtDNA content was determined using real time quantitative PCR analysis [32] on ABI sequence detection system 7700 (Applied Biosystems) and was expressed as ratio of copy number of mtDNA to copy number of β2 microglobulin (β*2M*) gene (nDNA). The mtDNA/nDNA ratio in tumor was divided by the ratio in the surrounding non-cancerous tissue to obtain the ratio of mtDNA level in tumor to that in normal tissue. The ratio of >1 means that the mtDNA content in the tumor is higher than that in the surrounding tissue. The ratio of <1 means that the mtDNA content is reduced in the tumor when compared to its surrounding non-cancerous tissue.

### Statistical analysis

Fisher's exact test is used to analyze whether there is association between the mtDNA content change in tumors and the presence of mutations. Non-parametric Mann-Whitney test was also used to test if mtDNA content alteration in tumor is different in patients with or without mtDNA mutations. P-values less than 0.05 are considered statistically significant.

## Results

### Somatic mtDNA mutations in esophageal primary tumors

**Figure 1:**
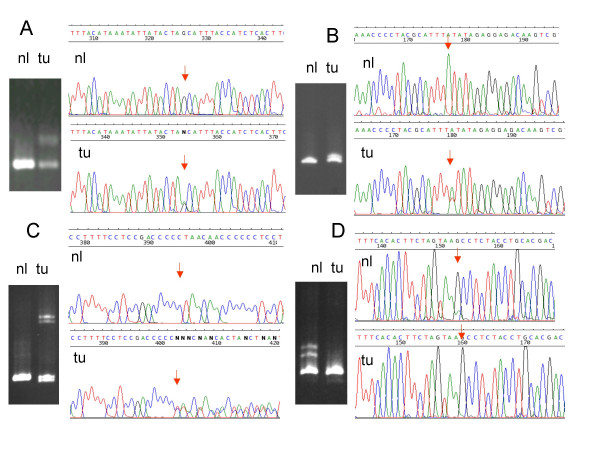
**Novel somatic mtDNA mutations in esophageal cancer detected by TTGE and sequencing. **A: Homoplasmic to heteroplasmic G10500A (A11T in ND4L) mutation in case E12 B: Heteroplasmic change of A1544T in 12S rRNA in case E05 C: Heteroplasmic deletion of 11bp (del TAACAACCCCC)at np10941 (110 × in ND4) in case E18 D: Heteroplasmic to homoplasmic change of A9182G (N219S) in ATPase 6 in case E15. The multiple banding pattern of TTGE in tumor confirms heteroplasmic change. tu: tumor, nl: normal

To test if somatic mtDNA mutation is a general phenomenon in esophageal cancer and to characterize the mutation spectrum, the TTGE mutation detection method was used to screen the entire mitochondrial genome of tumor and surrounding normal tissues. Figure 1 depicts the four novel somatic mtDNA mutations. Panels A-C each showed single band to multiple band change from surrounding normal tissue to tumor tissue indicating a homoplasmy to heteroplasmy alteration, while panel D showed an alteration from a heteroplasmy to homoplasmy. Direct sequencing of the DNA fragment allowed the identification of the mutations as shown in Figure 1 and listed in Table 1. Eleven out of 20 (55%) tumors harbored somatic mtDNA mutations with a total of 14 mutations. Nine mutations were found in the D-loop region, one in 12S rRNA and four in mRNA. Eleven tumors were found to have somatic mutations (Table 1). All nine mutations in the D-loop involved an insertion or deletion within the poly C stretch of the nucleotide positions (np) 303–309 hot spot region. One of them, the T at nucleotide position 310 was changed to C, resulting a homopolycytidine stretch of 15 C's. The long stretch of poly C might increase the instability of this region. Two of the somatic mutations changed from the homoplasmic state in surrounding tissue to a mutant homoplasmic state in tumor. Three changed from heteroplasmic state in normal to homoplasmic state in tumor, and 6 changed from homoplasmic wild type to heteroplasmic mutant. Three had heteroplasmy in both the surrounding tissue and the tumor, but the proportions of the mutant mtDNA in the paired tissues are different.

### Germline sequence variations

When the sequence of surrounding tissue was compared with revised Cambridge reference mtDNA sequence deposited in GenBank (accession No: NC_001807) and MitoMap , numerous germline sequence variations were found. A total of 185 distinct germline variations have been identified from the sequenced fragments. These do not represent all the sequence variations in the specimens analyzed since only the DNA regions that show somatic mutations, either homoplasmic or heteroplasmic, by TTGE were sequenced. Ten of these variations are novel (Table 2), and 175 (data not shown) of them have been recorded in the Mitomap database. Eight of these novel variations occurred in mRNA region, and five were missense variations (Table 2). Among them, the most remarkable is A11976C in NADH dehydrogenase subunit 4 (ND4) where tyrosine at amino acid position 406 is replaced by serine. Other novel missense variations are M47T in NADH dehydrogenase subunit 4 (ND4L), S531T in NADH dehydrogenase subunit 5 (ND5), S219N in ATP synthase subunit 6 (ATPase 6), and E109D in cytochrome c oxidase subunit II (COII). The reported polymorphisms are mostly unremarkable. Other frequent germ-line polymorphisms are C16233T, T16189C, T16519C in D-loop region, and C12705T in ND5, and T9540C in cytochrome c oxidase subunit II (COII) are silent variations with no amino acid change.

### Significance of novel somatic and germline mutations

**Figure 2:**
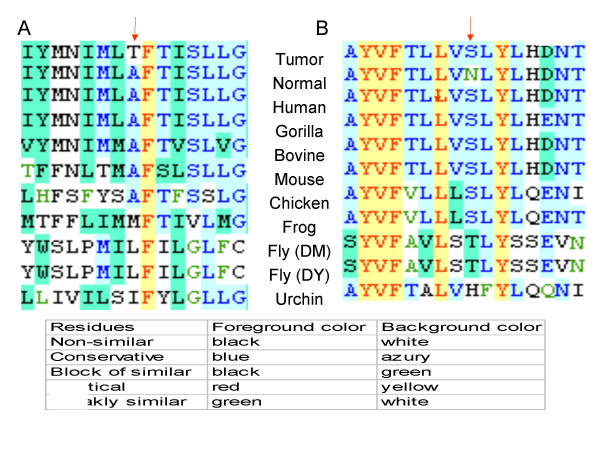
**Alignment of novel somatic missense mutations. **A: Case E12, G10500A mutation (A11T in ND4L) and B: Case E15, A9182G (N219S in ATPase 6)

The frameshift mutation, 10941del TAACAACCCCC, causing frame-shift and premature termination at amino acid 110 of ND4, is expected to be a deleterious mutation. It involves a homoplasmy to heteroplasmy change. G10500A somatic mutation changes a moderately conserved alanine residue to threonine in ND4L (Fig. 2A). The A9182G mutation in ATPase6 is a back change from asparagine to serine. The wildtype is serine, but it is asparagine in the patient's normal tissue and changes back to serine in the tumor. The amino acid is located in a moderately conserved region (Fig. 2B). One silent mutation G9377A in COIII (cytochrome c oxidase subunit III) does not change the amino acid. However, it does not exclude the possibility that the nucleotide substitution may cause impairment in RNA processing due to improper precursor RNA folding. Another mutation, A1544T in 12S rRNA occurs at a stem region. This mutation causes the disruption of a critical base pair. It may destabilize the rRNA structure.

**Figure 3:**
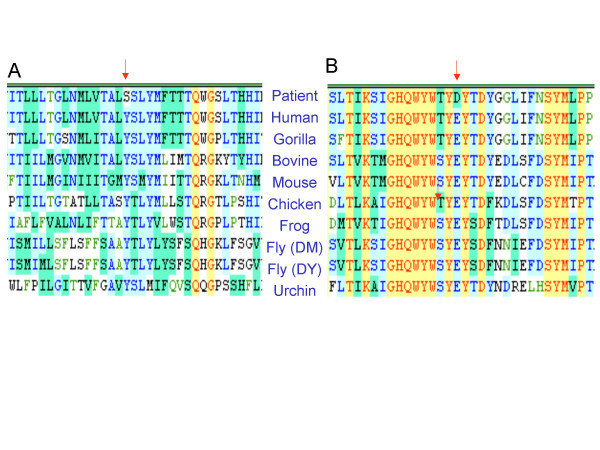
**Alignment of novel germline missense mutations **A: Case E02, A11976C (Y406S in ND4) and B: Case E16, G7921C (E109D in COII)

In addition to somatic mtDNA mutations, novel missense germline mutations may also cause affect on mitochondrial function. For instance, A11976C changes an evolutionary highly conserved hydrophobic tyrosine to hydrophilic serine at amino acid position 406 of ND4 subunit (Fig. 3A), would potentially cause functional effect. Another example is the E109D mutation in COII (Fig. 3B). Although it involves a conserved amino acid change, the shortening of hydrocarbon chain may have subtle effects on mitochondrial function. Accumulation of the subtle effect over time may increase an individual's risk of developing cancer. All five novel missense germline variations were not detected in 19 breast cancers, 31 neurofibromas, and 15 medulloblastomas, all of Caucasian origin in previous studies [7,11,13]. They were also not present in 18 oral cancers and 20 liver cancers of Taiwanese patients [8,12].

### Molecular and clinicopathological characteristics

To investigate if the down-regulation of mitochondrial function in cancers is due to regression of mtDNA biogenesis, the relative amount of mtDNA in tumor compared to that in the surrounding non-cancerous tissue was measured by real-time quantitative PCR analysis. Table 3 lists the results and clinicopathological characteristics of each tumor. The mean value of tumor/normal mtDNA ratio in the tumors with mtDNA mutation was 1.32 ± 0.31 and that of the tumors without mtDNA mutation was 1.45 ± 0.22. Thus, tumor with or without somatic mtDNA mutations show no significant difference (*p *value = 0.59 using non-parametric Mann-Whitney test) in mtDNA content change from normal to tumors. The majority (6 out of 11) of tumors with somatic mtDNA mutations have either elevated (>2) or reduced (<0.5) mtDNA content in tumors compared to surrounding normal tissue. However the *p *value (0.27) does not reach a statistically significant level as analyzed by Fisher's exact test. Analysis of a larger sample size will be required. The patient (case E18) whose mtDNA content is severely reduced in tumor harbored a frameshift deleterious mutation (10941 del 11bp). He was also the youngest patient (38 years of age). His tumor was at stage III and metastasized to lymph nodes. He expired 12 months after surgery. The patient (case E18) had a previous history of tongue cancer. The patient (case E05) who had the most reduced mtDNA content in tumor, harbored the A1544T novel mutation in 12S rRNA gene. His tumor was at stage III and had metastasized. He expired 4 months after the surgery. These two cases are consistent with the possibility that mtDNA mutations are associated with impaired biogenesis of mitochondrial genome. Four tumors had more than 2 fold increase in mtDNA content. Two of them (case E10 and E17) had 303–309 hot spot poly C mutation. Both patients had metastatic tumors, and died post-surgery. Two other patients had small non-metastatic tumors.

**Figure 4:**
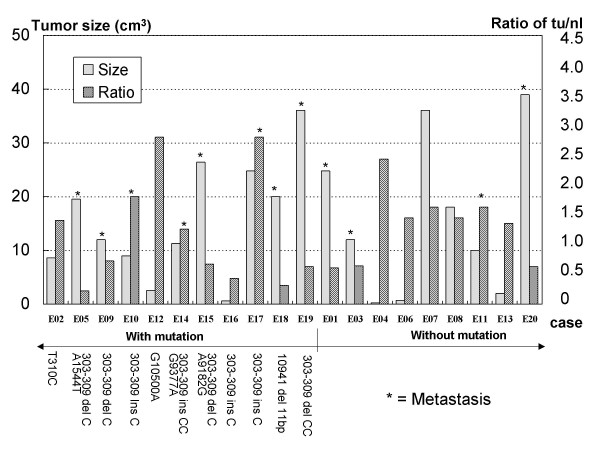
Histogram of mtDNA content change and tumor size with metastatic status of tumor.

The relationship of the mtDNA content changes in tumor with tumor size, and metastatic status is depicted in figure 4. The majority of tumors display an inverse relationship between tumor size and mtDNA content change (Fig. 4). Eight out of 11 tumors with mtDNA mutations became metastatic, whereas only 4 out of 9 tumors without somatic mtDNA mutations became metastatic. The analysis of odds ratio showed that the patients with somatic mtDNA mutation has 3.33 times higher risk of metastasis compared to patients without somatic mutations. However, due to a small sample size, it does not reach the statistic significant level (odds ratio: 3.33, 95% CI: 0.515–21.6). In order to reach statistical significance, the study of a larger sample size will be necessary.

## Discussion

This study represents the first mutational scanning of the entire mitochondrial genome of esophageal cancer. We screened the entire mitochondrial genome of 20 esophageal primary tumors and their surrounding tissues for the presence of somatic mutations using the TTGE mutation detection method. Fourteen somatic mtDNA mutations were found in 11 (55%) tumors.

The specificity and sensitivity of TTGE in screening both nuclear and mitochondrial DNA mutations have been evaluated in detail [29,30]. In general, TTGE can detect low levels of heteroplasmy that cannot be detected by direct DNA sequencing. Although the sensitivity of detecting nuclear gene mutations has been reported to be around 97%, the detection rate for mtDNA may be lower due to low percentage of heteroplasmy and the limit of DNA sequencing [29,30,33]. Thus, the mutations detected represent an underestimate of the total somatic mutation load in the tumor mtDNAs. Although homoplasmy to homoplasmy change was reported as the most frequent mtDNA alterations in tumors, the majority of esophageal cancers harbored heteroplasmic changes. The observation of heteroplasmic to heteroplasmic or heteroplasmic to homoplasmic changes and back changes from surrounding normal to tumor tissues suggests that mtDNA alterations may have already occurred before the pathological changes can be detected [6]. These results, although contradicted with the previous report that majority of the mutations involved in homoplasmy to homoplasmy changes, were consistent with random occurrence of mtDNA alterations at different times during tumorigenesis, or, mutations that might become homoplasmic at different rate. The mutant mitochondria may be present at either homoplasmic or heteroplasmic state depending on the number of cell division they have gone through, and their replicative advantage. Heteroplasmic mutations have been found in other tumors [8,11,12]. Thus, histologically normal surrounding tissues may not be normal at molecular level. In this regard, peripheral blood may be a better choice. Our results suggest that mtDNA alterations may potentially be good biomarkers for early detection and/or prognosis of cancer.

Frequency of mutations in the non-coding D-loop region is high, but the missense and frameshift mutations found in the coding region are the ones likely to cause an effect on mitochondrial function. Mitochondrial DNA depletion may not be the general mechanism for reduced mitochondrial oxidative phosphorylation activity in tumors as it has been reported [34,35]. Both somatic mutations and altered mtDNA content could be responsible for the down-regulation of bioenergetic function of mitochondria observed in most tumors [20]. In this study, the number of tumors with increased mtDNA content is just about equal to the number of tumors containing reduced mtDNA. This phenomenon was also observed in liver cancer where tumors bearing somatic mtDNA mutation had either markedly reduced or elevated mtDNA content in tumor [12]. It is not clear if the down-regulation of mitochondrial function by somatic mutation or reduced mitochondrial biogenesis is the cause or result of tumorigenic growth. In the cases where increased mtDNA content in tumor was observed, it could be explained by the amplification of mtDNA to compensate for mitochondrial dysfunction. This compensatory mechanism may be beneficial as observed in patients E04 and in mitochondrial disease [36].

In this study, several novel germline missense mutations were identified. Although the biochemical consequence of homoplasmic polymorphisms are considered too subtle to cause any detectable effect on oxidative phosphorylation, long term accumulation of the subtle difference in oxidative phosphorylation activity may eventually result in oxidative stress. Thus, in the late onset of a disease such as cancer, mtDNA polymorphisms can potentially play a role in modifying the cancer risk. The missense variations, Y406S in ND4, M47T in ND4L, S219N in ATPase 6, and E109D in COII, might function as cancer predisposition factors. Our unpublished observations have demonstrated that certain mtDNA haplogroups and mtDNA variations (polymorphisms and mutations) modify an individual's cancer risk. In order to understand the actual functional effect of the mtDNA mutations on susceptibility to cancer and tumorigenic process, functional analysis of mitochondrial bearing such mutations or polymorphisms in a transmitochondrial cybrid system will be necessary.

## Conclusion

We used TTGE to scan the entire mitochondrial genome for somatic mtDNA mutations in esophageal cancer, followed by DNA sequencing to identify the exact mutations and found that 55% of the tumors harbored somatic mtDNA mutations. Several novel missense mutations at both germline and somatic levels are likely to have an effect on mitochondrial function. Since reduction in mtDNA content is not the only mechanism leading to down-regulation of mitochondrial function in cancer, the possibility that a mutant protein generated by somatic mtDNA mutation plays a role in tumor formation can not be ruled out.

The results from this study indicate the importance of complete analysis of the entire mitochondrial genome when the functional significance of somatic mtDNA alterations in cancer is to be investigated. In addition to qualitative alterations, quantitative changes in mtDNA content are also important markers responsible for down-regulation of mitochondrial function in cancer. Both germline and somatic mtDNA mutations appeared to have effects in modifying cancer risk or are directly involved in the pathogenic mechanism of tumorigenesis.

## Competing interests

The author(s) declare that they have no competing interests.

## Authors' contributions

DJT performed the TTGE and sequencing analyses, analyzed the data, and prepared tables and figures for the manuscript.

LLL assisted in sequencing analysis.

RKB performed the real time quantitative analysis of the mitochondrial DNA copy number.

JC collected the tissue specimens and the clinical data, examined the pathology of the tissue, and prepared the pathology reports.

YFW isolated the DNA from the tissue specimens.

KTY supervised the pathology studies.

LJW designed the study project, interpreted the data, drafted and led the completion of the manuscript and gave the final approval of the version for publication.

All authors read and approved the final manuscript.

## Pre-publication history

The pre-publication history for this paper can be accessed here:



## Figures and Tables

**Table 1:** Somatic mtDNA mutations in esophageal cancer

**Case**	**Gene**	**Tu vs Nl**	**Pattern**	**Codon**	**AA change**	**Comment**	**Frequency in normal population (Genbank) (%)**
E02	D-loop	T310C, 303–315 ins C (C15)	HT→ HT			polymorphism	16/1622 (0.99)
E05	D-loop	303–309 del C (C8→ C7/8)	HM→ HT			polymorphism	71/1622 (4.38)
E05	12S	**A1544T**	HM→ HT			**novel**	**0/2460 (0)**
E09	D-loop	303–309 del C (C9/8→ C8/9)	HT→ HT			polymorphism	71/1622 (4.38)
E10	D-loop	303–309 ins C (C8→ C8/9)	HM→ HT			polymorphism	50/1622 (3.08)
E12	ND4L	**G10500A**	HM→ HT	GCA→ ACA	**A11T**	**novel**	**0/**1622 (0)
E14	D-loop	303–309 ins CC (C7→ C9/10)	HM→ HT			polymorphism	24/1622 (1.48)
E14	COIII	G9377A (back change)	HM→ HM	TGG→ TGA	W57W	polymorphism	42/2460 (1.71)
E15	D-loop	303–309 del C (C8/9→ C8)	HT→ HM			polymorphism	71/1622 (4.38)
E15	ATPase 6	**A9182G **(back change)	HT→ HM	AAC→ AGC	**N219S**	**novel**	**0/2460 (0)**
E16	D-loop	303–309 ins C (C7/8→ C7/8/9)	HT→ HT			polymorphism	50/1622 (3.08)
E17	D-loop	303–309 ins C (C8→ C9)	HM→ HM			polymorphism	50/1622 (3.08)
E18	ND4	**10941 del TAACAACCCCC**	HM→ HT	frameshift	**110X**	**novel**	**0/2460 (0)**
E19	D-loop	303–309 del CC (C9/10→ C7)	HT→ HM			polymorphism	24/1622 (1.48)

12S:12S ribosomal RNA, ND4L: NADH dehydrogenase subunit 4L, ND4:NADH dehydrogenase subunit 4, ATPase 6:ATP synthase F0 subunit 6, COIII: Cytochrome C oxidase subunit III

HT: Heteroplasmy; HM: Homoplasmy, AA: Amino Acid, Tu: tumor, Nl: Normal

Missense and novel mutations are in bold

Total mutation: 14, hm→ ht: 6, ht→ hm:3, hm→ hm: 2, ht→ ht: 3

**Table 2:** Novel germ-line sequence variations in esophageal cancer

**Case**	**Gene**	**Change**	**Pattern**	**Position**	**Codon**	**AA change**	**Frequency in this study**	**Frequency in normal population (Genbank) (%)**
E01	ND5	T12957C	Hm	12957	AAT→ AAC	N207N	1	9/2461 (3.66)
E02	ND4	A11976C	Ht	11976	TAC→ TCC	**Y406S**	1	0/2461 (0)
E03	Dloop	C481T	Hm	481			1	3/1901 (0.16)
E08	ND5	G12561A	Hm	12561	CAG→ CAA	Q75Q	1	4/2461 (0.16)
E13	D-loop(mtTF1 binding site)	G251A	Hm	251			1	0/1624 (0)
E13	ND4L	T10609C	Hm	10609	ATA→ ACA	**M47T**	1	44/2461 (1.79)
E13	ND5	G13928C	Hm	13928	AGC→ ACC	**S531T**	1	78/2461 (3.17)
E15	ATPase 6	G9182A	Ht	9182	AGC→ AAC	**S219N**	1	4/2461 (0.16)
E16	COII	G7912C	Hm	7912	GAG→ GAC	**E109D**	1	0/2461 (0)
E19	COII	T7711C	Hm	7711	CTT→ CTC	L42L	1	8/2461 (0.32)

MtTF1: mitochondrial transcription factor, ND5:NADH dehydrogenase subunit 5, COII: Cytochrome c oxidase subunit II

Missense mutations are in bold

**Table 3:** The somatic mtDNA mutations and clinicopathological characteristics of esophageal tumors.

Case	Sex	Age (Yrs)	Tumor Size (cm^3^)	TNM	Survival	Stage	mtDNA tu/nl Ratio	Number of Mutation	Mutation
E01	M	42	24.75	T3N1M0	Alive, 51 month post surgery	III	0.67	0	
E02	F	57	8.6	T3N0M0	Alive, 48 month post surgery	IIA	1.56	1	T310C, 303→ 315 ins C
E03	M	46	12	T2N1M0	10 month	IIB	0.71	0	
E04	M	65	0.2	T1N0M0	Alive, 48 month post surgery	I	2.7	0	
E05	M	50	19.5	T3N1M0	4 month	III	0.25	2	303–309 del C (C8→ C7/8), A1544T
									
E06	M	70	0.65	T2N0M0	Alive, 4 month	IIA	1.6	0	
E07	M	52	36	T3N0M0	13 month	IIA	1.8	0	
E08	M	62	18	T4N0M0	12 month	III	1.6	0	
E09	M	66	12	T3N1M0	12 month	III	0.8	1	303–309 del C (C9/8→ C8/9)
E10	M	73	9	T3N1M0	6 month	III	2.0	1	303–309 ins C (C8→ C8/9)
E11	M	68	10	T2N1M0	34 month	IIB	1.8	0	
E12	M	51	2.55	T1N0M0	Alive, 60 month post surgery	I	3.1	1	G10500A
E13	M	52	2	T1N0M0	3 month	I	1.5	0	
E14	M	60	11.25	T3N1M0	7 month	III	1.4	2	303–309 ins CC(C7→ C9/10), G9377A
									
E15	M	67	26.4	T3N1M0	5 month	III	0.75	2	303–309 del C, A9182G
									
E16	M	68	0.06	T2N0M0	Alive, 38 month post surgery	IIA	0.48	1	303–309 ins C (C7/8→ C7/8/9)
E17	M	61	24.75	T2N1M0	20 month	IIB	3.1	1	303–309 ins C (C8→ C9)
E18	M	38	20	T4N1M0	12 month	III	0.35	1	10941del TAACAACCCCC
E19	M	68	36	T2N1M0	14 month	IIB	0.7	1	303–309 del CC (C9/10→ C7)
E20	M	61	39	T3N1M0	8 month	III	0.7	0	

TNM: tumor-node-metastasis classification
